# Study protocol for the health economic evaluation of outpatient long-term video EEGs for people with seizure disorders alongside the ALVEEG study – a randomized controlled equivalence trial

**DOI:** 10.1186/s12913-025-12738-1

**Published:** 2025-04-18

**Authors:** Ricarda Sophia Schulz, Pauline Sarah Münchenberg, Carmen Thomas, Alice Charlotte Dorison, Hans-Aloys Wischmann, Ana Sofia Oliveira Gonçalves, Imke Mayer, Kerstin Wainwright, Martin Holtkamp, Christian Meisel, Tobias Kurth, the ALVEEG Consortium, Bernd Vorderwülbecke, Mirja Steinbrenner, Matthias Endres, Claudia Gorski, Fabian Prasser, Angela Kaindl, Bernhard Weschke, Cornelia Potratz, Pascal Fenske, Felix von Podewils, Astrid Bertsche, Sarah Mai Viebahn, Bernadette Gaida, Norbert Utzig, Juliane Schulz, Thomas Mayer, Peter Hopp, Nils Holert, Miriam Wienecke, Georg Leonhardt, Peggy Müller, Petra Knobelsdorf, Antke Wolter, Anne Klinker, Mara Brandebusemeyer, Uwe Nussbaum, Jannis Seemann

**Affiliations:** 1 Institute of Public Health, Charité – Universitätsmedizin Berlin, Berlin, Germany; 2 Owkin Ltd, London, UK; 3 Computational Neurology, Department of Neurology, Charité – Universitätsmedizin Berlin, Berlin, Germany; 4 Computational Neurology, Berlin Institute of Health, Charité – Universitätsmedizin Berlin, Berlin, Germany; 5 Center for Stroke Research Berlin, Charité – Universitätsmedizin Berlin, Berlin, Germany; 6 NeuroCure Cluster of Excellence, Charité – Universitätsmedizin Berlin, Berlin, Germany; 7 Bernstein Center for Computational Neuroscience, Berlin, Germany

**Keywords:** Seizure disorders, Epilepsy, Healthcare management, Outpatient long-term video EEGs, Statutory health insurance, Equivalence trial, Health economic evaluation, Claims data, Cost-effectiveness, Cost-utility

## Abstract

**Background:**

Epilepsy and other seizure disorders are medical conditions that impose a substantial health economic burden on society given their considerable costs of illness and use of healthcare resources. The ALVEEG trial aims to tackle resource shortages in clinical settings and optimize patient management by evaluating outpatient ambulatory long-term video electroencephalograms (ALVEEGs) as a new diagnostic pathway to diagnose and manage epilepsy and other seizure disorders. The health economic evaluation alongside this trial aims to determine the cost-effectiveness and cost-utility of ALVEEGs for affected patients presenting themselves at participating epilepsy centers in Germany.

**Methods:**

This study protocol comprises the rationale and methods of the health economic evaluation of ALVEEGs embedded into the ALVEEG project. We will perform cost-effectiveness and cost-utility analyses, with the outcomes being a priori defined endpoint measures of the main trial. We will consider the proportion of solved clinical queries (primary endpoint), the number of hospital stays, the in-patient length of stay, and quality-adjusted life years for the here presented health economic evaluation. Costs will be collected by the participating health insurance companies alongside the trial, with the evaluation being conducted from a statutory health insurance perspective within the German healthcare system. The reporting of the economic evaluation follows the Consolidated Health Economic Evaluation Reporting Standards (CHEERS) checklist.

**Discussion:**

The health economic evaluation of ALVEEGs for patients affected by epilepsy and other seizure disorders within the German healthcare system will deliver insightful evidence on the cost-effectiveness and cost-utility of the intervention and hence guide policy and decision makers regarding a potential inclusion of ALVEEGs into the health benefit basket of the statutory health insurance scheme.

**Trial registration:**

German Clinical Trials Register (DRKS00032220), date registered: December 11, 2023.

## Introduction

### Background

Epilepsy and other seizure disorders, such as psychogenic non-epileptic seizures or syncopes, are medical conditions that affect all ages and impose a substantial health economic burden on society given their considerable costs of illness and use of healthcare resources [1, 2]. In 2019, the illness-specific costs for common neurological disorders amounted to €17.2 billion in Germany, of which €1.6 billion (9.3%) were attributable to patients with epilepsy or epileptic seizures according to the German Federal Statistical Office (*Statistisches Bundesamt (Destatis)*) [3]. Over the last years, epilepsy-specific costs of illness in patients with epilepsy have increased, mainly due to higher indirect costs [3, 4].

An accurate and timely diagnosis of epilepsy and other seizure disorders is crucial to create a correct treatment plan, to prevent severe or even fatal seizures, and to reduce associated costs [5]. However, long-term video-electro-encephalogram (LVEEG) monitoring in Germany is currently only available in specialized inpatient settings, requiring significant time efforts and resources [6]. In comparison, other countries such as the United States of America, the United Kingdom and Australia have already established outpatient long-term video-electroencephalograms (ALVEEGs) to decrease costs and increase availability and scalability, thus shortening time to diagnosis, as well as improving patient satisfaction, while being cheaper compared to inpatient video telemetry [7, 8].

With the enactment of the Digital Healthcare Act (*Digitale-Versorgung-Gesetz, DVG*) in 2021, the promotion of telehealth services in Germany has been significantly increased [9]. The study presented here aims to evaluate if ALVEEGs, using wearable sensor technologies and artificial intelligence (AI) to synthesize data from the electroencephalograms (EEGs) as basis for the neurologist’s diagnosis or treatment plan, in a home setting in selected eastern states of Germany as a new diagnostic pathway, are equally effective as the current inpatient-monitoring gold standard to diagnose and manage epilepsy and other seizure disorders. One special feature of the new diagnostic pathway is the use of artificial AI to analyze EEGs for diagnosis.

As with every new intervention, it is important to weigh the outcomes of ALVEEGs against their associated costs in comparison to standard care by conducting a health economic evaluation. To this day, the costs associated with an ALVEEG are not covered by the statutory German healthcare system [10]. To investigate the economic impact of ALVEEGs on the German healthcare system, a health economic evaluation will be conducted alongside the ALVEEG trial, including cost-effectiveness analyses and a cost-utility analysis from a statutory health insurance perspective. Findings from this economic evaluation will provide high-quality evidence for cost-effectiveness and will guide decision-makers including ALVEEGs into the health benefit basket of the German statutory health insurance scheme.

## Methods

### Trial design and study population

The ALVEEG study is a prospective, multicenter, randomized controlled equivalence trial with a duration of 27 months, carried out in selected eastern states in Germany. The main aim of the study is to improve the care of people with epilepsy and other seizure disorders by providing access to ALVEEGs in their own homes, and thus establishing a new form of care, bearing the potential to be highly relevant for diagnosing and managing such disorders as well as for the medical infrastructure.

Complete information on the study design and primary study population is provided elsewhere [11]. In short, the study is conducted in cooperation with five German epilepsy centers in the states of Berlin, Brandenburg, Mecklenburg-West Pomerania, and Saxony, where patients are continuously recruited for the study. Patients in the intervention group (IG) are provided with the ALVEEG telemonitoring system at home, which uses AI to synthesize the data of the EEGs as basis for the neurologist’s recommendation, and will also have access to a smartphone app to document seizure activity, amongst other functions. The control group (CG) will receive care as usual, i.e., inpatient LVEEG monitoring and a paper diary for documentation. The following statutory health insurers are involved: BARMER, DAK-Gesundheit, and Techniker Krankenkasse (TK).

Eligible epilepsy centers had to (1) be led by neurologists/neuropediatrics/epileptologists, (2) meet the technical and spatial requirements for conducting inpatient LVEEG, and (3) treat patients who are insured within the statutory health system.

Patients are eligible to participate if they (1) have either experienced at least one seizure in the last 12 months prior to enrollment, or present with an established diagnosis of epilepsy, (2) have an indication 1–4 from the German procedure classification 1–210 (*Operationen- und Prozedurenschlüssel, OPS*), (3) are living in a home where they have access to 4G/LTE or faster wireless service, (4) are covered by the German statutory health insurance, and (5) present at one of the participating epilepsy centers. Patients can only be included if written informed consent is provided by them or by their legal representative.

Eligibility criteria for the population (patients) and setting (epilepsy centers) are listed in Table 1.

**Table 1:** Eligibility criteria population (patients) and settings (epilepsy centers) applied within the ALVEEG study

**Inclusion criteria**
** Population (patients)**
(1) All ages (0 years and older)
(2) At least one seizure in the last 12 months prior to enrollment, or an established diagnosis of epilepsy
(3) Indication 1–4 from the German procedure classification 1–210 (OPS)
(4) Living in a home with access to 4G/LTE or faster wireless service
(5) Covered by German statutory health insurance
(6) Present themselves at one of the participating epilepsy centers
** Setting (epilepsy centers)**
(1) Led by neurologists/neuropediatrics/epileptologists
(2) Fulfill the technical, organizational and spatial requirements for carrying out long-term inpatient video EEGs
(3) Treat patients with statutory health insurance
**Exclusion criteria**
** Population (patients)**
(1) Indication for presurgical epilepsy diagnostics, i.e., indication 5 from German procedure classification 1–210 (OPS)
(2) Additional psychosocial needs
(3) Privately insured or self-paying
(4) Lack of informed consent or lack of informed consent of the legal representative
**Setting (epilepsy centers)**
(1) Located outside the catchment area (Eastern states of Germany: Berlin, Brandenburg, Mecklenburg-Western Pomerania, and Saxony)
(2) Failure to meet technical, organizational and spatial requirements
(3) Do not treat patients of the statutory health insurance system

*4G* Fourth-generation wireless, *EEG* Electroencephalogram, *OPS* German procedure classification (*Operationen- und Prozedurenschlüssel*)

ALVEEG's primary endpoint is the proportion of clinical questions resolved in the intervention group (IG) versus the CG. A clinical question is considered resolved if (1) a diagnosis specification is required and determined by LVEEG, (2) treatment management needs to be defined or reassessed and is determined with the help of the LVEEG, or (3) a diagnosis specification and a reassessment and adaptation of the treatment management are required and are both determined by the LVEEG.

For the health economic evaluation presented here, we will consider data on the following endpoints: Proportion of solved clinical queries (primary endpoint), quality of life (QoL) and number and cumulative length of hospital stays as outcomes.

### Sample size

Our working hypothesis for the health economic evaluation is that the intervention is equivalent to the comparator (SLVEEG) both clinically and in terms of incurred costs. Therefore, we use a two-sided t-test to conduct inference. The presented sample size calculation was based on the study’s primary endpoint, i.e., the proportion of clinical questions resolved in the IG versus the CG. For LVEEGs, the proportion of resolved clinical cases is expected to be 0.8 (80%) [12]. Equivalence of diagnostic success is defined in a relative margin of ± 15% deviation from the proportion in the CG. To reach a power of 90% for detecting a significant result at a level of alpha = 5%, in a Z-test comparing two proportions, a sample size of 482 patients is required. Taking an expected dropout rate of 30% into account, the required sample size sums up to 688 participants.

Details on the sample size calculation for the main trial are detailed elsewhere [11].

In the context of the ALVEEG trial, only claims data of patients insured by one of the participating statutory health insurances can be analyzed. We assume a latency period for claims data of up to 9 months for outpatient claims data and up to 6 months for inpatient claims data, typically observed in the German healthcare setting. As a consequence, we will not be able to analyze data of all participants enrolled in the ALVEEG study who are insured with the aforementioned insurances. The final data extraction for the claims data is scheduled for March 2027.

Counting backward, considering the 9-month latency period and the patient-individualized 3-month follow-up period, we will have complete primary and claims data for patients recruited until March 2026, when about 4/5 of the trial will be completed. We assume that patients are recruited uniformly throughout the study. Therefore, we expect that by March 2026, we will have recruited 80% of the target sample size. As patients are randomized equally to IG and CG, half of them will be in the IG, and the other half in the CG, resulting in 550 participants at that time point.

### Effects/outcomes

For the cost-effectiveness analyses, we will consider (1) the proportion of patients with solved clinical queries, which is the primary endpoint of the ALVEEG study, as well as the endpoints: (2) number of hospital stays, and (3) cumulative length of hospital stay (days) per hospitalized patient as outcome measures. We will treat hospital stays and their length as non-exhaustive measures for the participant’s health status, as well as epilepsy management, patient’s awareness of their disease, and conditions possibly caused by epilepsy-related events. Patient-specific information will be derived through data collected alongside the treatment process and delivered by the participating health insurance companies.

The outcome measure for the cost-utility analysis will be quality-adjusted life years (QALYs). They will be measured and calculated using the European Quality of Life Questionnaire – 5 Dimensions (EQ- 5D- 5L), filled out by participants at study entry and at the follow-up visit 3 months after the discussion of diagnostic findings. The EQ- 5D- 5L is a patient-reported outcome measure comprising five dimensions, namely mobility, self-care, usual activities, pain and discomfort, and anxiety and depression, conceptualized as a tool to measure QoL of patients independently of their illness [13].

To calculate the QALYs, we will apply the area under the curve approach, considering the baseline and the follow-up assessment scores. We will use German population utility weights and control our analyses for baseline scores [14].

For an overview of the outcomes of the main trial, see Table 2.

**Table 2:** Information on endpoints of the main trial considered as outcomes for the health economic evaluation of the ALVEEG study

Outcomes	CG	IG	Time points
**Primary outcome**			
Proportion of clinical questions resolved in the IG versus the CG	X	X	At discussion of medical findings (patient, neurologist)
**Secondary outcomes**			
# hospital stays	X	X	Starting between enrollment and 3 months after discussion of medical findings
Cumulative length of hospital stays for hospitalized patients	X	X	Starting between enrollment and 3 months after discussion of medical findings
Quality of Life	X	X	At baseline and 3 months after discussion of medical findings

*CG* Control group, *IC* Intervention group

### Costs

We will consider the following cost categories in our analyses: Direct medical costs, direct non-medical costs, and indirect costs. Direct medical costs include costs arising due to hospital stays, ambulatory care, medication, as well as medical aids and appliances. Direct non-medical costs will comprise reimbursed travel costs to medical examinations, while sick pay will be considered as indirect costs. It has to be noted that typically sick leave payments are only provided by statutory health insurances after the 42nd day of sickness. Prior to this, expenses are borne by employers in the German healthcare system and therefore excluded from our analysis. Due to the objectives of the ALVEEG study, costs for at-home nursing care, care services, and home help will not be considered. The same applies to intangible costs from the perspective of the health insurance. The observational period is patient-individualized from randomization into the study until 3 months after the discussion of medical findings. Cost data will be delivered by the participating health insurance companies.

An overview of considered costs for the economic evaluation of the study is displayed in Table 3.

**Table 3:** Considered costs for the health economic evaluation of the ALVEEG study

Cost category	Costs considered	CG	IG	Data source
**Claims data delivered by the participating health insurance companies**				Health insurance claims data*
** Direct costs**				
Medical costs	Inpatient services	X	X	
	Outpatient services	X	X	
	Prescriptions for drugs	X	X	
	Therapeutic appliances	X	X	
	Assistive devices	X	X	
Non-medical costs	Patient transport services	X	X	
** Indirect costs**	Sickness leave benefits	X	X	
**Investment costs**	Outpatient long-term video-EEG devices (ALVEEGs)		X	Provided by manufacturer
**Maintenance costs**	Costs arising due to maintenance services for the ALVEEGs		X	Provided by manufacturer
**Operating costs**	Costs for training related to the usage of ALVEEGs per time spent		X	Tariff contracts, surveys
	Staff costs for the practical application of ALVEEGs in clinical practice		X	Tariff contracts, surveys

*CG* Control group, *IG* Intervention group

*Participating health insurance companies: BARMER, DAK-Gesundheit, and Techniker Krankenkasse

All costs will be presented in Euros (€) and we will set 2026 as the reference year. We will apply a left-censoring approach for this health economic evaluation, meaning that we will only include those services and costs into our analysis, for which the start date fell within the trial period. For the sickness leave benefits, the reference data will be the start date of the sickness leave, not the start date of the payments. Because of the randomized design of the study, we assume that the selection of this specific approach will have the same effect on both, IG and CG.

We will compare the costs and effects for both groups over a duration of 3 months post discussion of diagnostic findings. Due to this relatively short time period under consideration, costs and outcomes will not be discounted as the Institute for Quality and Economy in Health Services (*Institut für Qualität und Wirtschaftlichkeit im Gesundheitswesen, IQWiG*) suggests.

After the assessment of costs and collected outcome data, we will compute incremental cost-effectiveness ratios (ICERs), the difference in the mean costs between IG and CG divided by the difference in the mean outcomes between those groups.

In addition to the patient-specific costs, we will consider recurring costs for operating and maintaining the equipment as well as depreciation for any investment costs incurred, including costs arising from the conduct of training sessions for the medical device and software applications for the staff members at the participating epilepsy centers, and costs for setting up the required infrastructure.

The intervention costs will be measured over the entire year, as considering a one-year period avoids seasonal effects [15].

## Statistical analysis

### Descriptive analysis

We will present baseline characteristics of the BARMER, DAK-Gesundheit, and TK patients, stratified by treatment group (IG and CG).

Mean values (standard deviation) for normally distributed continuous data, medians and interquartile ranges for non-normally distributed data, and proportions of frequencies for categorical data will be presented. Additionally, information on cost and health service utilization will be presented descriptively.

### Inferential analysis

We will use the parametric G-formula to estimate incremental costs and incremental effects [16].

​To analyze the costs for each group (control and intervention), zero-inflated gamma regression models will be fitted. The models will be adjusted for age and sex as well as for random effects for the epilepsy centers to account for inter-center variability using an Identity-link in order to determine the absolute cost differences (risk differences).

We will apply a similar procedure to calculate the incremental effects using the same adjustment set as for the cost models. For the cost-effectiveness analyses considering the primary endpoint as the outcome parameter, we assume a binomial distribution, while we assume a (zero-inflated) Poisson distribution of hospital stays. The binomial models will be applied with a logit-link. For the cumulative length of hospital stays (cost-effectiveness), we assume a zero-inflated gamma distribution and add an identity-link to the model.

The regression model with QALYs as the dependent variable will further be adjusted for the EQ- 5D- 5L baseline score as this is highlighted as important in the literature [14], and differences in differences will be calculated. We will apply linear models (normal distribution) for this cost-utility-analysis.

Finally, the ICERs will be calculated for the different analysis approaches.

### Handling of missing values

Patients with missing values for the primary endpoint will be excluded from the analyses in line with the statistical analyses of the main trial. For the other considered endpoints, those patients with missing values for the respective endpoint will be further excluded following an available case analysis approach. Assuming a missing at random mechanism, we will impute missing values for the adjustment variables applied in the regression models by multiple imputations for each arm of the study, if any adjustment variables are missing [17].

With respect to the cost-utility analysis, it is probable that the values for the different EQ- 5D- 5L domains at baseline and at the follow-up assessment will show different patterns of missing data [18]. The consequence of missing values at one of the assessment time points would mean that the aggregated QALY information would also be missing. Given the cumulative nature of QALYs, they take different levels of aggregation (dimensions, scores, QALYs). In the context of the present analysis, we will first impute the individual domains of the EQ- 5D- 5L and the EQ- 5D- 5L scores and the QALYs will subsequently be imputed passively.

### Handling uncertainty

To address uncertainties arising throughout our analyses, we will use multiple techniques and statistical procedures such as bootstrapping and multiple imputation (for example). ​In addition, both linear models (LMs) and generalized linear models (GLMs) with a gamma distribution and identity-link will be employed for cost regressions. Furthermore, mixed-effects variants of these models, specifically linear mixed-effects models (LMER) and generalized linear mixed-effects models (GLMER) as implemented in the glmmTMB R-package [19] will be applied.

Further, applying bootstrapping allows us to summarize the uncertainty due to sampling variation without making parametric assumptions about the distribution. We will use the generated bootstrap interactions to create cost-effectiveness diagrams to visually present the joint distribution of incremental costs and effects as well as the uncertainty of cost-effectiveness results.

To obtain 95% confidence intervals (CIs) for the incremental costs and effects, we will then combine bootstrap inference and multiple imputation. For the calculation of the CIs, we will apply the boot MI method [20], i.e., a nonparametric bootstrapping approach with 5,000 replications. The 2.5% and 97.5% percentiles of the resulting distribution of the average metric (over 5 imputed datasets) will be considered as CIs for each of the resulting bootstrap datasets after multilevel imputation.

The reporting of the health economic evaluation of the ALVEEG study will adhere to the guidelines outlined in the Consolidated Health Economic Evaluation Reporting Standards (CHEERS) [21]. All statistical analyses will be conducted using the statistical software R [22] version 4.4.1 (or more recent) and RStudio.

## Discussion

Epilepsy and other seizure disorders impose a substantial economic burden on society, with increasing illness-specific costs. The diagnosis and treatment management of these disorders require better access and care for patients, especially in rural areas, where there is often a lack of resources and neurological experts. Furthermore, an overall shortage of inpatient LVEEG monitoring capacities must be addressed, especially given the ongoing staffing challenges in hospitals [23–25]. By harnessing the availability of innovative, wearable LVEEG diagnostics and AI to synthesize data, the ALVEEG study addresses these issues, offering the possibility of an outpatient gold standard epilepsy diagnosis for children, adolescents, and adults in their home environment. The goal is to optimize cross-sectoral care, indirectly reduce morbidity, and lower costs as well as to enable a new form of diagnosis and care that is highly relevant for epilepsy patients and the medical infrastructure.

To establish the link between the outcomes of ALVEEGs and its associated costs, a health economic evaluation will be conducted alongside the trial. An intervention is generally considered cost-effective if the ICER falls below a certain threshold. However, today there is no such threshold in Germany. Despite this, we plan to present the cost-utility and cost-effectiveness results in a way that allows decision-makers to take an informed decision whether or not to adopt the intervention.

The generated evidence could be useful in the epilepsy medical care field in comparable health care systems. However, it may not be generalizable to healthcare settings outside of Germany.

Our planned health economic evaluation has the following strengths: First, we consider the perspective of statutory health insurance funds, receiving the patient-specific costs directly via the claims data of the participating statutory health insurances. Therefore, the data will not be biased by patients’ self-reported information, which might be influenced, e.g., by recall bias and possible misclassifications. Second, with respect to the outcomes, the usage of the EQ- 5D- 5L to calculate utility weights is another strength, as it is a widely used and valid generic instrument, so that our results will be comparable with other similar studies. Third, we will address the skewness of cost data and methodological uncertainties by applying advanced statistical modelling techniques and sensitivity analyses. Lastly, we will consider different outcome parameters in our analyses, so we can provide a more multifaceted view of the aspects of the intervention.

However, there are some limitations to the health economic evaluation, which need to be considered when interpreting the results. Given the above-mentioned latency period for up to 9 months, we will not be able to analyze claims data from patients whose enrollment date will be close to the end of the trial, because their claims data will not be available to the participating health insurers. We expect that this will impact the CG and IG in the same way, given the study design. Moreover, we will only be able to analyze claims data for patients insured by BARMER, DAK-Gesundheit, or TK, the participating three statutory health insurances. However, although in total, there are 96 statutory health insurances in Germany, the insurance companies participating in this trial are part of the largest type of German statutory health insurances (*Verband der Ersatzkassen*) [26]. Finally, while our patient follow-up period is sufficient to capture the near-term costs and outcomes analyzed in our study, it is not long enough to fully assess the long-term impact of the interventions.

## Conclusion

By providing a widely available and cost-effective way to diagnose and manage epilepsy and other seizure disorders, the ALVEEG trial aims to prevent a lack of healthcare services and challenges the current shortage of inpatient video-EEG monitoring capacities, especially in rural, often underserved areas. The results of the economic evaluation conducted alongside this study will deliver high-quality nuanced evidence for decision-makers and policy implications. Furthermore, the insights gained from this evaluation will be invaluable for further out-patient telemonitoring initiatives that can optimize the care provision for patients with epilepsy and other seizure disorders.

## Data Availability

No datasets were generated or analysed during the current study.

## Abbreviations

AI: Artificial Intelligence
ALVEEGs: Outpatient long-term video-electroencephalograms
CG: Control group
CIs: Confidence Intervals
EEG: Electroencephalogram
EQ- 5D- 5L: European Quality of Life Questionnaire – 5 Dimensions
GLM: Generalized Linear Model
GLMM: Generalized Linear Mixed Model
ICER: Incremental Cost-Effectiveness Ratio
IG: Intervention group
IQWiG: Institute for Quality and Economy in health Systems; *Institut für Qualität und Wirtschaftlichkeit im Gesundheitswesen*
LVEEG: Long-term video-electroencephalogram
OPS: *Operationen**- und **Prozedurenschlüssel**;* German Procedure Classification
QALYs: Quality-adjusted life years
QoL: Quality of life
TK: Techniker Krankenkasse
4G/LTE: Fourth Generation mobile communications standard/Long Term Evolution
